# Supplementary material for: Creating interventions to transition long-lasting insecticide net distribution in Ghana
Source: BMJ Open. 2022 Jun 1;12(6):e063121. doi: 10.1136/bmjopen-2022-063121 (PMC9161095; doi:10.1136/bmjopen-2022-063121)
Supplement: Author's manuscript [file bmjopen-2022-063121.draft_revisions.pdf]

BMJ Open

BMJ Open is committed to open peer review. As part of this commitment we make the peer review history of every article we publish publicly available.

When an article is published we post the peer reviewers' comments and the authors' responses online. We also post the versions of the paper that were used during peer review. These are the versions that the peer review comments apply to.

The versions of the paper that follow are the versions that were submitted during the peer review process. They are not the versions of record or the final published versions. They should not be cited or distributed as the published version of this manuscript.

BMJ Open is an open access journal and the full, final, typeset and author-corrected version of record of the manuscript is available on our site with no access controls, subscription charges or pay-per-view fees (http://bmjopen.bmj.com).

If you have any questions on BMJ Open's open peer review process please email info.bmjopen@bmj.com

BMJ Open

Creating Interventions to Transition Long Lasting Insecticide Net Distribution in Ghana

Journal:	*BMJ Open*
Manuscript ID	bmjopen-2022-063121
Article Type:	Original research
Date Submitted by the Author:	23-Mar-2022
Complete List of Authors:	Glozah, Franklin; University of Ghana, Social and Behavioural Sciences<br>Asampong, Emmanuel; University of Ghana, Social and Behavioural Sciences<br>Tabong, Philip ; University of Ghana, Social and Behavioural Sciences<br>Nwameme, Adanna; University of Ghana<br>HORNUVO, RUBY; University of Ghana, Social and Behavioural Sciences<br>Chandi, Margaret; Ghana Health Service<br>Peprah, Nana; Ghana Health Service<br>Adongo, Philip; University of Ghana<br>Dako-Gyeke, Phyllis; University of Ghana School of Public Health, Department of Social and Behavioural Sciences
Keywords:	Public health < INFECTIOUS DISEASES, PUBLIC HEALTH, Tropical medicine < INFECTIOUS DISEASES, SOCIAL MEDICINE

SCHOLARONE™
Manuscripts

I, the Submitting Author has the right to grant and does grant on behalf of all authors of the Work (as defined in the below author licence), an exclusive licence and/or a non-exclusive licence for contributions from authors who are: i) UK Crown employees; ii) where BMJ has agreed a CC-BY licence shall apply, and/or iii) in accordance with the terms applicable for US Federal Government officers or employees acting as part of their official duties; on a worldwide, perpetual, irrevocable, royalty-free basis to BMJ Publishing Group Ltd ("BMJ") its licensees and where the relevant Journal is co-owned by BMJ to the co-owners of the Journal, to publish the Work in this journal and any other BMJ products and to exploit all rights, as set out in our licence.

The Submitting Author accepts and understands that any supply made under these terms is made by BMJ to the Submitting Author unless you are acting as an employee on behalf of your employer or a postgraduate student of an affiliated institution which is paying any applicable article publishing charge ("APC") for Open Access articles. Where the Submitting Author wishes to make the Work available on an Open Access basis (and intends to pay the relevant APC), the terms of reuse of such Open Access shall be governed by a Creative Commons licence – details of these licences and which Creative Commons licence will apply to this Work are set out in our licence referred to above.

Other than as permitted in any relevant BMJ Author's Self Archiving Policies, I confirm this Work has not been accepted for publication elsewhere, is not being considered for publication elsewhere and does not duplicate material already published. I confirm all authors consent to publication of this Work and authorise the granting of this licence.

For peer review only

Creating Interventions to Transition Long Lasting Insecticide Net Distribution in Ghana

Franklin N. Glozah[1]; Emmanuel Asampong[1]; Philip Teg-Nefaah Tabong[1]; Ada Nwameme[1]; Ruby Hornuvo[1]; Gloria. M. Chandi[2]; Nana Yaw Peprah[2]; Philip B. Adongo[1] Phyllis Dako-Gyeke[1]

[1]*Department of Social and Behavioural Sciences, School of Public Health, University of Ghana*
[2]*Ghana Health Service*

*Correspondence: Emmanuel Asampong
Email: easampong@uh.edu.gh

For peer review only

Abstract

Objective: Mass Long Lasting Insecticide Net (LLIN) distribution campaigns are rolled out, as a part of the Ghana Malaria Strategic plan (2021-2025) which seeks to protect at least 80% of the population at risk with effective malaria prevention interventions. Although, the mass LLIN distribution campaign indicates a comprehensive stakeholder engagement approach, it does not systematically transition into the basic primary health care structures within the Ghana Health Services. This paper presents the process and outcome of creating an innovative social intervention, which focuses on community mobilization and capacity building of Community Health Officers.

Methods: This study employed a concurrent triangulation mixed methods approach conducted across six districts in Eastern and Volta regions, Ghana. Findings were synthesized, grouped, and further distilled to guide the participatory co-creation workshops. Co-creation involved participatory learning in action technique which is a practical, adaptive research strategy which enabled diverse groups and individuals to learn, work and act together in a co-operative manner.

Results: The results suggest the establishment of a Community Health Advocacy Team (CHAT). This would be necessary in efforts aimed at transitioning LLIN distribution campaign in communities. The role of the CHAT would be centred on key elements of community/social mobilisation and capacity building, all nested in a Social and Behaviour Change Communication strategies.

Conclusion: The research team is in the process of assessing the acceptability and feasibility of the CHAT intervention with all stakeholders in the various communities. Assessment of the effectiveness of the CHAT intervention would be done at a later time.

Key Messages

What is already known on this topic?

- Periodically, the National Malaria Control Programme rolls out Mass LLIN distribution campaigns in communities. However, it has not been embedded into the primary health care structures. This study seeks to co-create an intervention that hinges on community mobilization and Community Health Officers playing key roles in the distribution campaigns.

What this study adds

- This study suggests a Community Health Advocacy Team to provide support services to transition Long Lasting Insecticide Net distribution campaign within communities

How this study might affect research, practice or policy

- Long Lasting Insecticide Net distribution campaign would be transitioned into the Community-based Health Planning and Services, improve use of Long Lasting Insecticide Net within communities, thereby reduce malaria morbidity and mortality.

For peer review only

For peer review only - http://bmjopen.bmj.com/site/about/guidelines.xhtml

INTRODUCTION

Malaria continues to be endemic in most parts of Africa, including Ghana, causing millions of lives to be lost across age categories.[1,2] There was however progress between 2016 – 2019 where Ghana made significant progress in malaria control. Cases decreased by 32% (from 237 cases per 1000 of the population at risk to 161 cases), and deaths decreased 7% (from 0.4 per 1000 of the population at risk to 0.37).[3] Mass Long Lasting Insecticide Net **(**LLIN) distribution campaigns are rolled out, as a part of the Ghana Malaria Strategic Plan (2021-2025) which seeks to protect at least 80% of the population at risk with effective malaria prevention interventions. In spite of progress made in overall LLIN ownership, challenges still remain. The Ghana Malaria Indicator Survey shows 67% access to a LLIN, with 43% use.[4] The inference therefore is that people have not taken up LLIN despite the distribution campaign. Although these campaigns expose high proportions of the Ghanaian community to LLIN interventions, they hardly lead to desired health-related behaviours (i.e., LLIN use).

Some barriers to LLIN use have been documented to include distribution challenges where there is inadequate number of nets per household, limited Social and Behaviour Change Communication (SBCC) activities as well as lack of continuous malaria education.[5-8] At the community level, there is knowledge gap on malaria prevention, inability to hang LLINs in many households due to housing type and sleeping places. There is also misuse and repurposing of LLINs.[9-10] Health-worker challenges have also been reported to include lack of adequate training on community mobilization skills, minimal number of staff and lack of follow-up, community engagement, and supervision.[11-12]

Thus, ability to achieve desired outcomes from LLIN campaigns may require adoption of social innovative approaches which support behaviour change within communities.[13]

For peer review only - http://bmjopen.bmj.com/site/about/guidelines.xhtml

Social Innovation is described as a collective process enabling the generation of ideas by people who participate collaboratively to improve delivery strategies in the community or health facility.[14] The social objective emphasizes the engagement of concerned communities within which interventions will be diffused with innovative approaches meeting both social and medical needs.[14] Community directed programmes provide opportunities for government, health services, social actors and individuals to work closely with populations directly affected by diseases, especially infectious conditions. One such example is the Community-based Health Planning and Services (CHPS) program in Ghana, which seeks to ensure accessible, equitable, efficient and good quality health care services.[15] The CHPS concept involves the provision of door-to-door primary health care services to community members by trained nurses known as Community Health Officers (CHOs).[15-17] CHOs provide antenatal care, family planning, health education, outreach clinics for delivery of child welfare services, and school health services.

In order to create a people centered intervention, the project (Improving the Effectiveness of Mass Long Lasting Insecticide-treated Net Distribution Campaigns Through Community-based Health Planning and Services Programme in Ghana) seeks to leverage on the CHPS to ensure community involvement and ownership of the LLIN Mass Distribution Campaigns in Ghana. Although some community health workers (e.g., health volunteers, community health nurses) participate in the registration of households, list potential beneficiaries, and distribute LLINs during the campaigns, their involvement is paid for on a contractual basis. To ensure sustainability, the need to transition this campaign into CHPS has become imperative as the National Malaria Control Programme continues to rollout the 2021 campaign, amidst the COVID-19 pandemic. This paper presents the process and outcome of the co-creation of an innovative social intervention which would transition the Mass LLIN distribution campaign into CHPS by focusing on community mobilization and capacity building.

For peer review only - http://bmjopen.bmj.com/site/about/guidelines.xhtml

METHODS

Study site

A total of six communities, one community per district across two regions in southern Ghana, participated in the study. These were communities in districts where the 2021 Point Mass Distribution (PMD) campaigns of LLINs, are ongoing. PMD is one of the strategies adopted by the Ghana Health Service and its partners whereby only designated sites are noted for distribution of LLINs. This was to avoid possible biases with regard to community engagement by ensuring that components align with the timelines of the National Malaria Control Programme (NMCP) and the funder. The study was conducted in districts with the highest prevalence of malaria as reported in the District Health Information Management System (DHIMS2), a comprehensive web-based application for remotely compiling data across different levels of a health system into a central storage point: Ho West (Tsito-90%), Ho (Takla Hokpeta-75%) and Agortime Ziope (Kpetoe-100%) in the Volta Region; and Birim South (Apoli-94%), Achiase (Achiase-94%) and Abuakwa North (Kukurantumi-93%) in the Eastern Region (data source: District Health Information Management System 2 - DHIMS 2).

Patient and public involvement

Participants in this study were not involved in setting the research question or the outcome measures. However, they were closely involved in the creation of the CHAT. Participants were also involved during inauguration of CHAT which helped to motivate community involvement during the study.

For peer review only - http://bmjopen.bmj.com/site/about/guidelines.xhtml

Population and sample

The study population consisted of household heads, community leaders, Non-Governmental Organisation (NGO) representatives, Community Health Officers (CHOs), as well as officers from the National Malaria Control Programme (NMCP) and the Ghana Health Service (GHS).

Study Design

We employed a concurrent triangulation mixed methods research design, involving participatory approaches within an implementation research framework. A desk review, survey, In-Depth Interviews (IDIs), Key Informant Interviews (KIIs), Focus Group Discussions (FGDs), and Participatory Workshops (PWs) were organized concurrently, albeit at different times.

Data Collection

Desk Review: A desk review was done to identify documents (i.e., articles and reports) containing guidance and recommendations on effective strategies relating to Mass LLIN Distribution Campaigns adopting community-based approaches in Ghana and elsewhere. This involved a comprehensive literature search and review to identify relevant published and grey literature. A desk review guide was used to collect appropriate data by initially presenting these in an extraction sheet that outlined potential barriers, enablers, lessons learnt and recommendations from similar interventions. Documents included scholarly journals, Ministry of Health documents and reports, Ghana Health Service documents and reports, National Malaria Control Programme documents and reports, WHO/TDR documents, and reports, as well as documents and reports from the coalition of NGOs in health, an umbrella and

For peer review only

coordinating body overseeing activities of all registered NGOs and Community Based Organisations (CBOs) in the health sector of Ghana.

Focus Group Discussions (FGDs): FGDs (14) were organised to contextualize and explore the barriers to, and enablers of Mass LLIN Distribution Campaigns in the context of community mobilization, capacity building and SBCC. FGDs involved purposively selected household heads (4), caregivers of children under 5 (4), and Community Health Officers (6).

Key Informant Interviews (KIIs): A semi-structured interview guide was used to conduct ten KIIs with purposively selected NMCP and Ghana Health Service focal persons at the regional and district levels to assess LLIN campaign delivery processes.

Baseline survey: A baseline survey (N = 800) across the six districts was done to identify baseline parameters to be used for assessing the effectiveness of our co-created intervention. These parameters included LLINs ownership, usage (regular, occasional and non-user), malaria morbidity among children under five and pregnant women. Data were collected using REDCap software on android tablets.

Intervention Co-creation

Findings from the Desk Review, FGDs, KIIs, and baseline surveys were synthesized, and grouped according to relevance. This was then distilled and formed the basis for developing a participatory workshop guide aimed at co-creating a LLIN campaign intervention involving various stakeholders (i.e., investigators, NGO representatives, School Health Education Programme Coordinators, ANC nurses, Disease Control Officers, District Health Management Teams (DHMTs), CHOs, community leaders and opinion leaders). Six participatory workshops were conducted employing the participatory learning in action technique, which is a practical, adaptive research strategy that enables diverse groups and individuals to learn, work

For peer review only - http://bmjopen.bmj.com/site/about/guidelines.xhtml

and act together in a co-operative manner, to focus on issues of joint concern, identify challenges and generate positive responses in a collaborative and democratic manner.[15]

Findings from the participatory workshops (Figure 1) were further synthesized to co-create our intervention.

Ethical Approval

The protocol for this study was approved by the Ghana Health Service Ethics Review Committee (GHS-ERC: 002/06/21).

Data Analysis

As part of the statistical analyses, we initially performed correlation and frequency analyses to identify and prioritise elements in LLIN campaign processes to inform the content of our participatory workshop guide. Also, thematic framework analysis was used to identify emerging themes to guide the establishment of Community Health Advocacy Teams (CHAT), focusing on community mobilization, capacity building and SBCC (Figure 2). We used NCapture and query functions in the NVivo Programme to display data and findings in the form of word clouds for easy visualization.

RESULTS

Socio-demographic Characteristics of Participants

A total of 106 participants comprising 50 women and 56 men, aged between 20 and 77years were involved in the participatory workshops. Participants were mainly NGO representatives, School Health Education Teachers, ANC nurses, Disease Control Officers, DHMTs, CHOs, Community leaders/Assemblymen, and opinion leaders (Table 1). Participants for the PW were from Kukurantumi, Achiase and Apoli in the Eastern Region as well as HoKpeta, Tsito and Kpetoe from the Volta Region of Ghana.

For peer review only - http://bmjopen.bmj.com/site/about/guidelines.xhtml

Table 1: Socio-Demographic Characteristics of Participatory Workshop Participants.

Characteristic of Participants	Number of Participants
**Community of Residence**	
Kukurantumi	18
Achiase	20
Apoli	19
HoKpeta	17
Tsito	17
Kpetoe	15
**Total**	**106**
**Sex**	
Female	50
Male	56
**Total**	**106**
**Age**	
<20years	-
20-29years	10
30-39years	61
40-49years	18
50+years	17
**Total**	**106**
**Educational Level**	
Primary	1
JHS/Secondary/Middle School	17
Tertiary	88
**Total**	**106**
**Marital Status**	
Single	28
Married	77
Divorced/Widowed/Separated	1
**Total**	**106**
**Length of Stay in Community**	
<20years	84
20-29years	7
30-39years	4

For peer review only

40-49years	1
50+years	10
**Total**	**106**

Community Health Advocacy Team (CHAT)

Findings from this study suggested that the establishment of a Community Health Advocacy Team (CHAT) will be very important in facilitating LLIN distribution campaigns within communities in Ghana (Fig. 1). CHAT will be made up of significant actors whose influences are recognized within communities. They will include Community Health Officers, religious leaders, School Health Education Programme coordinators, assemblymen/women, community information officers, representatives from any of the security services, community-based organizations and traditional authorities. The anticipated role of CHAT would be three-pronged. These are community/social mobilization, capacity building and social and behaviour change communication which will lead to improved use of LLIN. We found that the CHAT meetings are to be best convened quarterly, preferably by a Community Health Officer. Although it was emphasized that CHAT's efforts would not be compensated, there was a strong opinion among study participants that CHAT members be motivated tangibly or intangibly. For example, that they are given parcels of land where they could build (tangible) or be acknowledged publicly during community events (intangible).

Community/Social Mobilization

The National Malaria Control Programme (NMCP) in Ghana through its focal persons reach out to community members during LLIN distribution campaigns. These efforts could however be complemented by the CHAT since their positioning in the community make them readily available to support the NMCP. For CHAT to play this role creditably, its members must ensure the maintenance of NMCP's programming and its benefits over time.

For peer review only - http://bmjopen.bmj.com/site/about/guidelines.xhtml

Capacity Building

Findings reveal that CHAT must be trained by the NMCP as part of their capacity building efforts. CHAT would therefore need to be trained along the themed capacity building areas (training, registration, SBCC, logistics, distribution, and supervision) of the NMCP. In addition, CHAT members would be given skill enhancing strategies in leadership, communication, and community mapping, as well as record keeping competencies.

Social and Behaviour Change Communication

The NMCP offers Social and Behaviour Change Communication (SBCC) as part of the LLIN distribution campaign efforts. This study however revealed that the SBCC efforts could be strengthened if CHAT is actively involved at different stages of the campaign. For example, there could be SBCC activities prior to the campaign, during and after the campaign. Some of the channels identified include the use of posters, community information centers, home visits, and in churches and mosques. This would ensure that communities are well sensitized before and after the campaign.

DISCUSSION

This study used a participatory process[18,19] to develop a framework (campaign transitioning strategy) that could transition LLIN distribution into the Community-based Health Planning and Services structure, which is the Primary Health Care system in Ghana. We actively involved various stakeholders to strengthen the development of an intervention that has the potential to systematically address real-world problems,[16] and to achieve sustainable outputs and impact [20,21] in malaria prevention and control. This would also help to achieve the global agenda of eliminating malaria.

For peer review only - http://bmjopen.bmj.com/site/about/guidelines.xhtml

From a practical point of view, this framework will shape the policy on LLINs distribution to ensure a continuous all year-round campaign on the regular use of LLINs. This would not only help address the challenges associated with periodic campaign rolled out during the PMD but ensure a reduction in the access to use gap. Another practical contribution of this framework would be the development of a guideline and training manuals on capacity building that support the transfer of CHAT to other communities. The use of local resources makes the intervention sustainable and easily integrated into the health care delivery system in Ghana. If adopted, the CHAT could become one of the flagship malaria prevention and control interventions which can contribute toward malaria elimination. The framework would also be suitable during public health emergencies that tend to disrupt facility-based service delivery.[22] Disruptions to malaria control programmes during public health emergencies have been linked to over 75 major resurgences arising from the 1930s through the 2000s.[23]

In summary, the present foundational study provides a framework for transitioning LLINs campaign into the primary health care system in LMIC settings. In doing so, it will add value to implementation science and practice concerning the scaling up and advancement of such strategies to address challenges in LLINs campaigns. The next step is for investigators to assess the acceptability and feasibility of the CHAT campaign transitioning intervention. We are mindful of an obvious limitation which will be to consider contextual issues (malaria prevalence, social and cultural) that may relate to particular communities.

For peer review only

For peer review only - http://bmjopen.bmj.com/site/about/guidelines.xhtml

AUTHORS' CONTRIBUTIONS

All authors participated in designing the study with NYP and GMC providing technical support. PBA revised the final protocol. FG, EA and PTT collected and analysed the quantitative data while PDG was in charge of qualitative data collection and analysis. AN carried out the desk review. RH revised and finalized the CHAT framework. All authors participated in the Participatory Workshops. All authors contributed to writing the manuscript and approved of the final draft.

Competing Interests

The authors declare that they have no competing interests.

Funding

This work received financial support from the Health Campaign Effectiveness Programme, which is funded by the Bill & Melinda Gates Foundation at The Task Force for Global Health.

Data Sharing

Data are available upon reasonable request

For peer review only

REFERENCES

1. Allain JP, Owusu-Ofori AK, Assennato SM, et al. Effect of Plasmodium inactivation in whole blood on the incidence of blood transfusion-transmitted malaria in endemic regions: the african investigation of the mirasol system (AIMS) randomised controlled trial. Lancet. 2016;387(10029):1753–1761.

2. Ernst KC, Erly S, Adusei C, et al. Reported bed net ownership and use in social contacts is associated with uptake of bed nets for malaria prevention in pregnant women in Ghana. Malar J. 2017;16(1):13.

3. World Health Organisation (2019). World Malaria Report 2020

4. GSS/GHS/ICF Macro. *National Malaria Indicator Survey*. (2019).

5. Worrall, E., Were, V., Matope, A., Gama, E., Olewe, J., Mwambi, D., ... & Niessen, L. W. (2020). Coverage outcomes (effects), costs, cost-effectiveness, and equity of two combinations of long-lasting insecticidal net (LLIN) distribution channels in Kenya: a two-arm study under operational conditions. BMC public health, 20(1), 1-16. https://doi.org/10.1186/s12889-020-09846-4

6. Scott, J., Kanyangarara, M., Nhama, A., Macete, E., Moss, W. J., & Saute, F. (2021). Factors associated with use of insecticide-treated net for malaria prevention in Manica District, Mozambique: a community-based cross-sectional survey. *Malaria journal*, *20*(1), 1-9. https://doi.org/10.1186/s12936-021-03738-7

7. Aberese-Ako, M., Magnussen, P., Ampofo, G. D., & Tagbor, H. (2019). Health system, socio-cultural, economic, environmental and individual factors influencing bed net use in the prevention of malaria in pregnancy in two Ghanaian regions. Malaria journal, 18(1), 1-13. https://doi.org/10.1186/s12936-019-2994-5

8. Baltzell, K., Harvard, K., Hanley, M., Gosling, R., & Chen, I. (2019). What is community engagement and how can it drive malaria elimination? Case studies and

For peer review only - http://bmjopen.bmj.com/site/about/guidelines.xhtml

stakeholder interviews. Malaria journal, 18(1), 1-11. https://doi.org/10.1186/s12936-019-2878-8

9. Opoku, R., Amoah, P. A., & Nyamekye, K. A. (2021). Householders' perception about sustaining the useful life of long-lasting insecticide-treated nets in Ghana. International Health, 13(1), 57-62.

10. Bannor, R., Asare, A. K., Sackey, S. O., Osei-Yeboah, R., Nortey, P. A., Bawole, J. N., & Ansah, V. (2020). Sleeping space matters: LLINs usage in Ghana. *Pathogens and Global Health*, *114*(5), 271-278. https://doi.org/10.1080/20477724.2020.1776920

11. Assan, A., Takian, A., Aikins, M., & Akbarisari, A. (2018). Universal health coverage necessitates a system approach: an analysis of Community-based Health Planning and Services (CHPS) initiative in Ghana. Globalization and health, 14(1), 1-10. https://doi.org/10.1186/s12992-018-0426-x

12. Malede, A., Aemero, M., Gari, S. R., Kloos, H., & Alemu, K. (2019). Barriers of persistent long-lasting insecticidal nets utilization in villages around Lake Tana, Northwest Ethiopia: a qualitative study. BMC public health, 19(1), 1-11. https://doi.org/10.1186/s12889-019-7692-2

13. Wakefield, M. A., Loken, B. & Hornik, R. C. Use of mass media campaigns to change health behaviour. *The Lancet* vol. 376 1261–1271 (2010).

14. Dako-gyeke, P., Amazigo, U. V, Halpaap, B. & Manderson, L. Social innovation for health : engaging communities to address infectious diseases. *BMC Infect. Dis. Poverty* **9**, 1–4 (2020).

15. Nyonator, F. K., Awoonor-Williams, J. K., Phillips, J. F., Jones, T. C. & Miller, R. A. The Ghana Community-based Health Planning and Services Initiative for scaling up

For peer review only - http://bmjopen.bmj.com/site/about/guidelines.xhtml

service delivery innovation. *Health Policy and Planning* vol. 20 25–34 (2005).

16. Binka, F. N., Nazzar, A. & Phillips, J. F. The Navrongo Community Health and Family Planning Project. *Stud. Fam. Plann.* **26**, 121–139 (1995).

17. Awoonor-Williams, J. K. *et al.* The Ghana essential health interventions program: a plausibility trial of the impact of health systems strengthening on maternal & child survival. *BMC Health Serv. Res.* **13**, S3 (2013).

18. Loewenson, R. *et al.* Raising the profile of participatory action research at the 2010 Global Symposium on Health Systems Research. *MEDICC Rev.* **13**, 35–8 (2011).

19. McConnell, T. *et al.* Coproduction for feasibility and pilot randomised controlled trials: learning outcomes for community partners, service users and the research team. *Res Involv Engag.* **4**, (2018).

20. Jagosh, J. *et al.* Uncovering the benefits of participatory research: implications of a realist review for health research and practice. *Milbank Q.* **90**, (2012).

21. Leask, C., Sandlund, M., Skelton, D. & Chastin, S. Co-creating a tailored public health intervention to reduce older adults' sedentary behaviour. *Heal. Educ J* **76**, 595–608. (2017).

22. Agyepong, I. A. A Systems View and Lessons from the ongoing Ebola Virus outbreak in West Africa. *Ghana Med. J.* **48**, 168–172 (2014).

23. Walker, P. G. T. *et al.* Malaria morbidity and mortality in Ebola-affected countries caused by decreased health-care capacity, and the potential effect of mitigation strategies: a modelling analysis. *Lancet Infect Dis.* **15**, 825–832 (2016).

For peer review only

For peer review only - http://bmjopen.bmj.com/site/about/guidelines.xhtml

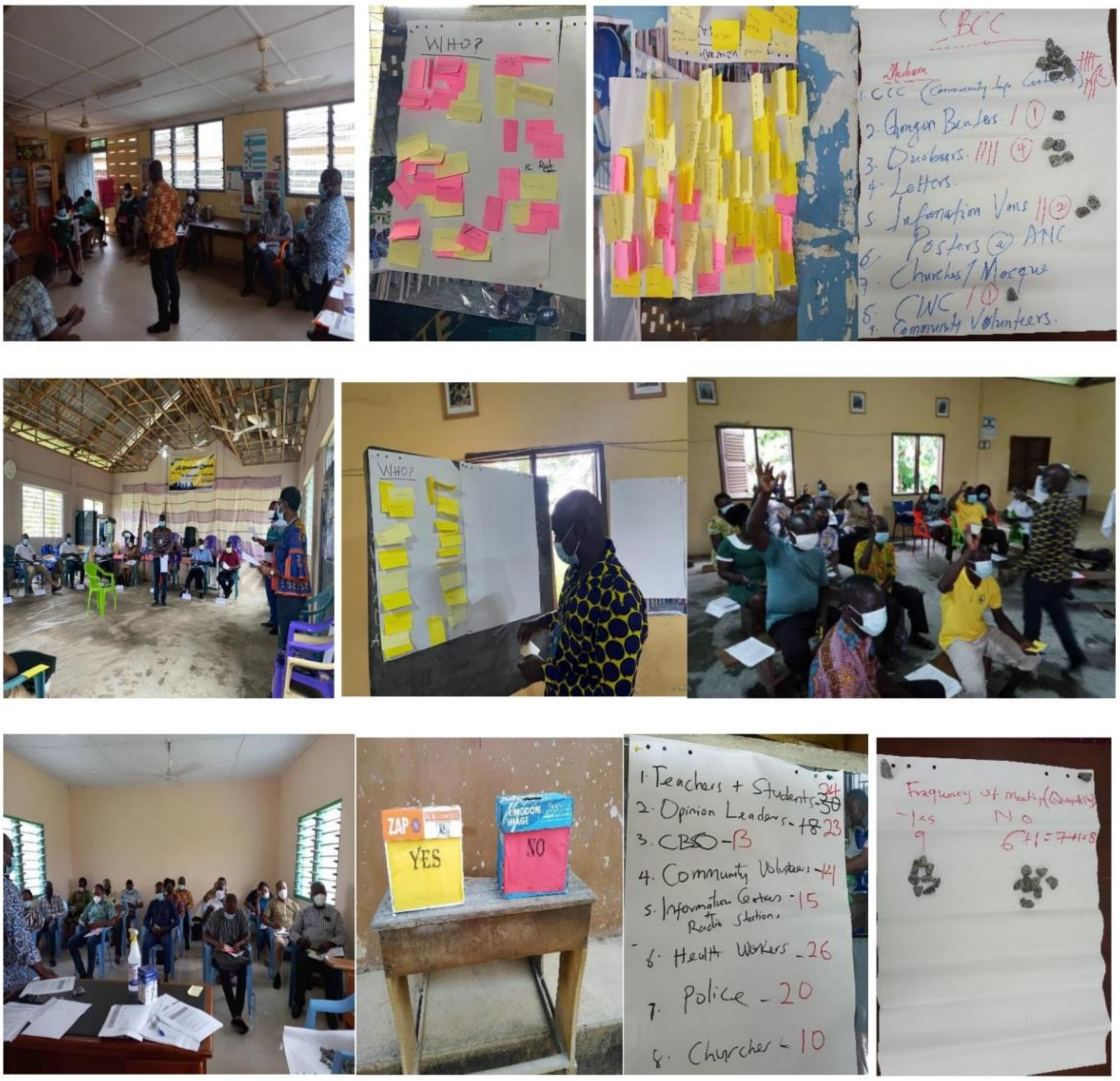

Figure 1: Images of Participatory Workshops held in the communities

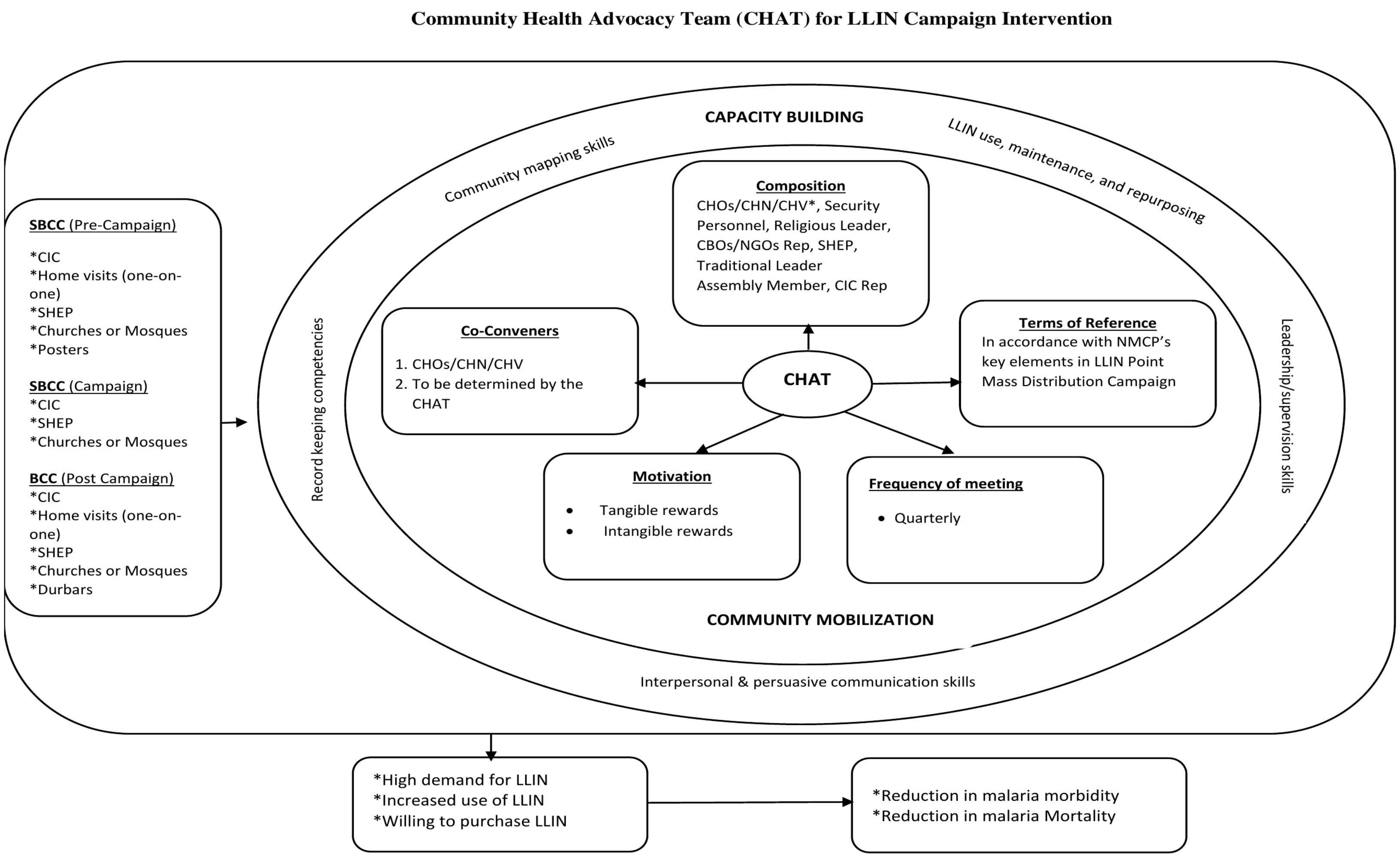

CHAT – Community Health Advocacy Team; SBCC – Social and Behaviour Change Communication; CIC – Community Information Centre; SHEP – School Health Education Programme; CHV – Community Health Volunteer, CHO – Community Health Officer; CHN – Community Health Nurse; CBO – Community Based Organization; NGO – Non-Governmental Organization

Figure 2: Community Health Advocacy Team (CHAT) for LLIN Campaign Intervention

APPENDIX

ITEM NO.	ITEM	DESCRIPTION	REPORTED ON PAGE NO.
1	Brief Name	The title or abstract identified of this social innovation in health research study.	1
2	Problem	Describe the current context, background and problem addressed by the social innovation from the perspective of the end user.	2
3	Rationale	Describe the rationale for the social innovation, including factors that show a change is needed from the perspective of the end user.	2-3
4	Social Innovation	Describe the key components of the social innovation. This could be accompanied by a detailed description, a photograph, or a figure. Describe each of the processes, activities, and elements used in the social innovation, including any enabling or supporting activities.	7-9
5	End Users	Describe the end users of the social innovation in health. Describe how end users are also direct or indirect beneficiaries of the social innovation.	8-9
6	Stakeholder Involvement	Describe how local stakeholders, including end users, are involved in design, development, implementation, and evaluation of the social innovation in health. In addition, describe the role of marginalized/vulnerable individuals or groups (e.g., people with disability or others as defined by the innovators) in these processes.	3-6
7	Inputs	Describe any physical, digital or informational materials used or distributed during training, delivery and/or implementation of in the social innovation; provide information on where the materials can be accessed† (e.g. online, appendix, URL).	5-6
8	Provider	For each category of the social innovation provider (e.g. community member, trained layperson, other individual), describe their expertise, background, role and any specific training given.	8-9
9	Implementation Strategy	Describe the implementation strategy for the social innovation and whether it is delivered individually, as a group, or partnership. Describe the level of external resources for implementation (e.g., internet access). Describe the frequency and duration of the social innovation delivery.	8-9

ITEM NO.	ITEM	DESCRIPTION	REPORTED ON PAGE NO.
10	Monitoring & Evaluation Strategy	Describe what is measured, how, and when as part of monitoring and evaluation. This includes measurement of health, social, and other impacts.	N/A
11	Setting	Describe the population, type(s) of location(s) where the social innovation is delivered, including any necessary social, political, cultural, environmental or other contextual issues. Describe at what level the innovation is implemented (e.g., district, subdistrict, village). This includes a description of the online setting for online social innovation.	N/A
12	Adaptability	Consider how the social innovation could be adapted, scaled up, or used in contexts other than the one described, if appropriate.	12
13	Financing	Describe how the social innovation in health has been funded at design, development, implementation, and evaluation stages. Describe how the social innovation could generate revenue (if applicable) or be institutionalized (if applicable) in order to be sustained in the future.	N/A
14	Health Impact	Describe the health impact of the social innovation over a period of time and the methods to assess health impact. Health is defined broadly here according to the WHO definition.	N/A
15	Social Impact	Describe the non-medical impact of the social innovation over a period of time. This could be impact on the environment, social changes, or other non-medical impact (e.g. lessons learned, new processes that emerged from the project, new relationships and networks, application of learned processes to other problems).	N/A
16	Limitations	Describe the limitations and potential unintended consequences of the social innovation in health during the design, development, or implementation.	12
17	Strengths	Describe how the social innovation in health improves on conventional practice.	12

SOCIAL INNOVATION IN HEALTH INITIATIVE

SESH Crowdsourcing For Health

TDR For research on diseases of poverty
UNICEF • UNDP • World Bank • WHO

For peer review only - http://bmjopen.bmj.com/site/about/guidelines.xhtml

Creating Interventions to Transition Long Lasting Insecticide Net Distribution in Ghana

Journal:	*BMJ Open*
Manuscript ID	bmjopen-2022-063121.R1
Article Type:	Original research
Date Submitted by the Author:	27-Apr-2022
Complete List of Authors:	Glozah, Franklin; University of Ghana, Social and Behavioural Sciences<br>Asampong, Emmanuel; University of Ghana, Social and Behavioural Sciences<br>Tabong, Philip ; University of Ghana, Social and Behavioural Sciences<br>Nwameme, Adanna; University of Ghana<br>HORNUVO, RUBY; University of Ghana, Social and Behavioural Sciences<br>Chandi, Margaret; Ghana Health Service<br>Peprah, Nana; Ghana Health Service<br>Adongo, Philip; University of Ghana<br>Dako-Gyeke, Phyllis; University of Ghana School of Public Health, Department of Social and Behavioural Sciences
**Primary Subject Heading**:	Infectious diseases
Secondary Subject Heading:	Health services research, Public health, Qualitative research
Keywords:	PUBLIC HEALTH, Tropical medicine < INFECTIOUS DISEASES, Health policy < HEALTH SERVICES ADMINISTRATION & MANAGEMENT, SOCIAL MEDICINE

SCHOLARONE™
Manuscripts

I, the Submitting Author has the right to grant and does grant on behalf of all authors of the Work (as defined in the below author licence), an exclusive licence and/or a non-exclusive licence for contributions from authors who are: i) UK Crown employees; ii) where BMJ has agreed a CC-BY licence shall apply, and/or iii) in accordance with the terms applicable for US Federal Government officers or employees acting as part of their official duties; on a worldwide, perpetual, irrevocable, royalty-free basis to BMJ Publishing Group Ltd ("BMJ") its licensees and where the relevant Journal is co-owned by BMJ to the co-owners of the Journal, to publish the Work in this journal and any other BMJ products and to exploit all rights, as set out in our licence.

The Submitting Author accepts and understands that any supply made under these terms is made by BMJ to the Submitting Author unless you are acting as an employee on behalf of your employer or a postgraduate student of an affiliated institution which is paying any applicable article publishing charge ("APC") for Open Access articles. Where the Submitting Author wishes to make the Work available on an Open Access basis (and intends to pay the relevant APC), the terms of reuse of such Open Access shall be governed by a Creative Commons licence – details of these licences and which Creative Commons licence will apply to this Work are set out in our licence referred to above.

Other than as permitted in any relevant BMJ Author's Self Archiving Policies, I confirm this Work has not been accepted for publication elsewhere, is not being considered for publication elsewhere and does not duplicate material already published. I confirm all authors consent to publication of this Work and authorise the granting of this licence.

For peer review only

Creating Interventions to Transition Long Lasting Insecticide Net Distribution in Ghana

Franklin N. Glozah[1]; Emmanuel Asampong[1]; Philip Teg-Nefaah Tabong[1]; Ada Nwameme[1]; Ruby Hornuvo[1]; Gloria. M. Chandi[2]; Nana Yaw Peprah[2]; Philip B. Adongo[1] Phyllis Dako-Gyeke[1]

[1]*Department of Social and Behavioural Sciences, School of Public Health, University of Ghana*
[2]*Ghana Health Service*

*Correspondence: Emmanuel Asampong
Email: easampong@ug.edu.gh

Abstract

Objective: Mass Long Lasting Insecticide Net (LLIN) distribution campaigns are rolled out, as a part of the Ghana Malaria Strategic plan (2021-2025) which seeks to protect at least 80% of the population at risk with effective malaria prevention interventions. Although, the mass LLIN distribution campaign indicates a comprehensive stakeholder engagement approach, it does not systematically transition into the basic primary health care structures within the Ghana Health Services. This paper presents the process and outcome of creating an innovative social intervention, which focuses on community mobilization and capacity building of Community Health Officers.

Methods: This study employed a concurrent triangulation mixed methods approach conducted across six districts in Eastern and Volta regions, Ghana. Findings were synthesized, grouped, and further distilled to guide the participatory co-creation workshops. Co-creation involved participatory learning in action technique which is a practical, adaptive research strategy which enabled diverse groups and individuals to learn, work and act together in a co-operative manner.

Results: The results suggest the establishment of a Community Health Advocacy Team (CHAT). This would be necessary in efforts aimed at transitioning LLIN distribution campaign in communities. The role of the CHAT would be centred on key elements of community/social mobilisation and capacity building, all nested in a Social and Behaviour Change Communication strategies.

Conclusion: The research team is in the process of assessing the acceptability and feasibility of the CHAT intervention with all stakeholders in the various communities. Assessment of the effectiveness of the CHAT intervention would be done at a later time.

For peer review only - http://bmjopen.bmj.com/site/about/guidelines.xhtml

Key Messages

What is already known on this topic?

- Periodically, the National Malaria Control Programme rolls out Mass LLIN distribution campaigns in communities. However, it has not been embedded into the primary health care structures. This study seeks to co-create an intervention that hinges on community mobilization and Community Health Officers playing key roles in the distribution campaigns.

What this study adds

- This study suggests a Community Health Advocacy Team to provide support services to transition Long Lasting Insecticide Net distribution campaign within communities

How this study might affect research, practice or policy

- Long Lasting Insecticide Net distribution campaign would be transitioned into the Community-based Health Planning and Services, improve use of Long Lasting Insecticide Net within communities, thereby reduce malaria morbidity and mortality.

For peer review only

For peer review only - http://bmjopen.bmj.com/site/about/guidelines.xhtml

INTRODUCTION

Malaria continues to be endemic in most parts of Africa, including Ghana, causing millions of lives to be lost across age categories.[1,2] There was however progress between 2016 – 2019 where Ghana made significant progress in malaria control. Cases decreased by 32% (from 237 cases per 1000 of the population at risk to 161 cases), and deaths decreased 7% (from 0.4 per 1000 of the population at risk to 0.37).[3] Mass Long Lasting Insecticide Net (LLIN) distribution campaigns are rolled out, as a part of the Ghana Malaria Strategic Plan (2021-2025) which seeks to protect at least 80% of the population at risk with effective malaria prevention interventions. In spite of progress made in overall LLIN ownership, challenges still remain. The Ghana Malaria Indicator Survey shows 67% access to a LLIN, with 43% use.[4] The inference therefore is that people have not taken up LLIN despite the distribution campaign. Although these campaigns expose high proportions of the Ghanaian community to LLIN interventions, they hardly lead to desired health-related behaviours (i.e., LLIN use).

Some barriers to LLIN use have been documented to include distribution challenges where there is inadequate number of nets per household, limited Social and Behaviour Change Communication (SBCC) activities as well as lack of continuous malaria education.[5-8] At the community level, there is knowledge gap on malaria prevention, inability to hang LLINs in many households due to housing type and sleeping places. There is also misuse and repurposing of LLINs.[9-10] Health-worker challenges have also been reported to include lack of adequate training on community mobilization skills, minimal number of staff and lack of follow-up, community engagement, and supervision.[11-12]

Thus, ability to achieve desired outcomes from LLIN campaigns may require adoption of social innovative approaches which support behaviour change within communities.[13]

For peer review only - http://bmjopen.bmj.com/site/about/guidelines.xhtml

Social Innovation is described as a collective process enabling the generation of ideas by people who participate collaboratively to improve delivery strategies in the community or health facility.[14] The social objective emphasizes the engagement of concerned communities within which interventions will be diffused with innovative approaches meeting both social and medical needs.[14] Community directed programmes provide opportunities for government, health services, social actors and individuals to work closely with populations directly affected by diseases, especially infectious conditions. One such example is the Community-based Health Planning and Services (CHPS) program in Ghana, which seeks to ensure accessible, equitable, efficient and good quality health care services.[15] The CHPS concept involves the provision of door-to-door primary health care services to community members by trained nurses known as Community Health Officers (CHOs).[15-17] CHOs provide antenatal care, family planning, health education, outreach clinics for delivery of child welfare services, and school health services.

In order to create a people centered intervention, the project (Improving the Effectiveness of Mass Long Lasting Insecticide-treated Net Distribution Campaigns Through Community-based Health Planning and Services Programme in Ghana) seeks to leverage on the CHPS to ensure community involvement and ownership of the LLIN Mass Distribution Campaigns in Ghana. Although some community health workers (e.g., health volunteers, community health nurses) participate in the registration of households, list potential beneficiaries, and distribute LLINs during the campaigns, their involvement is paid for on a contractual basis. To ensure sustainability, the need to transition this campaign into CHPS has become imperative as the National Malaria Control Programme continues to rollout the 2021 campaign, amidst the COVID-19 pandemic. This paper presents the process and outcome of the co-creation of an innovative social intervention which would transition the Mass LLIN distribution campaign into CHPS by focusing on community mobilization and capacity building.

For peer review only - http://bmjopen.bmj.com/site/about/guidelines.xhtml

METHODS

Study site

A total of six communities, one community per district across two regions in southern Ghana, participated in the study. These were communities in districts where the 2021 Point Mass Distribution (PMD) campaigns of LLINs, are ongoing. PMD is one of the strategies adopted by the Ghana Health Service and its partners' whereby only designated sites are noted for distribution of LLINs. This was to avoid possible biases regarding community engagement by ensuring that components align with the timelines of the National Malaria Control Programme (NMCP) and the funder. The study was conducted in districts with the highest prevalence of malaria as reported in the District Health Information Management System (DHIMS2), a comprehensive web-based application for remotely compiling data across different levels of a health system into a central storage point: Ho West (Tsito-90%), Ho (Takla Hokpeta-75%) and Agortime Ziope (Kpetoe-100%) in the Volta Region; and Birim South (Apoli-94%), Achiase (Achiase-94%) and Abuakwa North (Kukurantumi-93%) in the Eastern Region (data source: District Health Information Management System 2 - DHIMS 2).

Patient and public involvement

Participants in this study were not involved in setting the research question or the outcome measures. However, they were closely involved in the creation of the CHAT. Participants were also involved during inauguration of CHAT which helped to motivate community involvement during the study.

For peer review only - http://bmjopen.bmj.com/site/about/guidelines.xhtml

Population and sample

The study population consisted of household heads, community leaders, Non-Governmental Organisation (NGO) representatives, Community Health Officers (CHOs), as well as officers from the National Malaria Control Programme (NMCP) and the Ghana Health Service (GHS).

Study Design

We employed a concurrent triangulation mixed methods research design, involving participatory approaches within an implementation research framework. A desk review, survey, In-Depth Interviews (IDIs), Key Informant Interviews (KIIs), Focus Group Discussions (FGDs), and Participatory Workshops (PWs) were organized concurrently, albeit at different times.

Data Collection

Desk Review: A desk review was done to identify documents (i.e., articles and reports) containing guidance and recommendations on effective strategies relating to Mass LLIN Distribution Campaigns adopting community-based approaches in Ghana and elsewhere. This involved a comprehensive literature search and review to identify relevant published and grey literature. A desk review guide was used to collect appropriate data by initially presenting these in an extraction sheet that outlined potential barriers, enablers, lessons learnt and recommendations from similar interventions. Documents included scholarly journals, Ministry of Health documents and reports, Ghana Health Service documents and reports, National Malaria Control Programme documents and reports, WHO/TDR documents, and reports, as well as documents and reports from the coalition of NGOs in health, an umbrella and

For peer review only - http://bmjopen.bmj.com/site/about/guidelines.xhtml

coordinating body overseeing activities of all registered NGOs and Community Based Organisations (CBOs) in the health sector of Ghana.

Focus Group Discussions (FGDs): FGDs (14) were organised to contextualize and explore the barriers to, and enablers of Mass LLIN Distribution Campaigns in the context of community mobilization, capacity building and SBCC. FGDs involved purposively selected household heads (4), caregivers of children under 5 (4), and Community Health Officers (6).

Key Informant Interviews (KIIs): A semi-structured interview guide was used to conduct ten KIIs with purposively selected NMCP and Ghana Health Service focal persons at the regional and district levels to assess LLIN campaign delivery processes.

Baseline survey: A baseline survey (N = 800) across the six districts was done to identify baseline parameters to be used for assessing the effectiveness of our co-created intervention. These parameters included LLINs ownership, usage (regular, occasional and non-user), malaria morbidity among children under five and pregnant women. Data were collected using REDCap software on android tablets.

Intervention Co-creation

Findings from the Desk Review, FGDs, KIIs, and baseline surveys were synthesized, and grouped according to relevance. This was then distilled and formed the basis for developing a participatory workshop guide aimed at co-creating a LLIN campaign intervention involving various stakeholders (i.e., investigators, NGO representatives, School Health Education Programme Coordinators, ANC nurses, Disease Control Officers, District Health Management Teams (DHMTs), CHOs, community leaders and opinion leaders). Six participatory workshops were conducted employing the participatory learning in action technique, which is a practical, adaptive research strategy that enables diverse groups and individuals to learn, work

For peer review only

and act together in a co-operative manner, to focus on issues of joint concern, identify challenges and generate positive responses in a collaborative and democratic manner.[15]

Findings from the participatory workshops (Figure 1) were further synthesized to co-create our intervention.

[Insert Figure 1 here]

Ethical Approval

The protocol for this study was approved by the Ghana Health Service Ethics Review Committee (GHS-ERC: 002/06/21).

Data Analysis

As part of the statistical analyses, we initially performed correlation and frequency analyses to identify and prioritise elements in LLIN campaign processes to inform the content of our participatory workshop guide. Also, thematic framework analysis was used to identify emerging themes to guide the establishment of Community Health Advocacy Teams (CHAT), focusing on community mobilization, capacity building and SBCC (Figure 2). We used NCapture and query functions in the NVivo Programme to display data and findings in the form of word clouds for easy visualization.

[Insert Figure 2 here]

For peer review only - http://bmjopen.bmj.com/site/about/guidelines.xhtml

RESULTS

Socio-demographic Characteristics of Participants

A total of 106 participants comprising 50 women and 56 men, aged between 20 and 77years were involved in the participatory workshops. Participants were mainly NGO representatives, School Health Education Teachers, ANC nurses, Disease Control Officers, DHMTs, CHOs, Community leaders/Assemblymen, and opinion leaders (Table 1). Participants for the PW were from Kukurantumi, Achiase and Apoli in the Eastern Region as well as HoKpeta, Tsito and Kpetoe from the Volta Region of Ghana.

Table 1*:* Socio-Demographic Characteristics of Participatory Workshop Participants.

Characteristic of Participants	Number of Participants
**Community of Residence**	
Kukurantumi	18
Achiase	20
Apoli	19
HoKpeta	17
Tsito	17
Kpetoe	15
**Total**	**106**
**Sex**	
Female	50
Male	56
**Total**	**106**
**Age**	
<20years	-
20-29years	10
30-39years	61
40-49years	18
50+years	17
**Total**	**106**
**Educational Level**	
Primary	1
JHS/Secondary/Middle School	17

For peer review only

Tertiary	88
**Total**	**106**
**Marital Status**	
Single	28
Married	77
Divorced/Widowed/Separated	1
**Total**	**106**
**Length of Stay in Community**	
<20years	84
20-29years	7
30-39years	4
40-49years	1
50+years	10
**Total**	**106**

For peer review only

Community Health Advocacy Team (CHAT)

Findings from this study suggested that the establishment of a Community Health Advocacy Team (CHAT) will be very important in facilitating LLIN distribution campaigns within communities in Ghana (Figure 2). CHAT will be made up of significant actors whose influences are recognized within communities. They will include Community Health Officers, religious leaders, School Health Education Programme coordinators, assemblymen/women, community information officers, representatives from any of the security services, community-based organizations and traditional authorities. The anticipated role of CHAT would be three-pronged. These are community/social mobilization, capacity building and social and behaviour change communication which will lead to improved use of LLIN. We found that the CHAT meetings are to be best convened quarterly, preferably by a Community Health Officer. Although it was emphasized that CHAT's efforts would not be compensated, there was a strong opinion among study participants that CHAT members be motivated tangibly or intangibly.

For example, that they are given parcels of land where they could build (tangible) or be acknowledged publicly during community events (intangible).

Community/Social Mobilization

The National Malaria Control Programme (NMCP) in Ghana through its focal persons reach out to community members during LLIN distribution campaigns. These efforts could however be complemented by the CHAT since their positioning in the community make them readily available to support the NMCP. For CHAT to play this role creditably, its members must ensure the maintenance of NMCP's programming and its benefits over time.

Capacity Building

Findings reveal that CHAT must be trained by the NMCP as part of their capacity building efforts. CHAT would therefore need to be trained along the themed capacity building areas (training, registration, SBCC, logistics, distribution, and supervision) of the NMCP. In addition, CHAT members would be given skill enhancing strategies in leadership, communication, and community mapping, as well as record keeping competencies.

Social and Behaviour Change Communication

The NMCP offers Social and Behaviour Change Communication (SBCC) as part of the LLIN distribution campaign efforts. This study however revealed that the SBCC efforts could be strengthened if CHAT is actively involved at different stages of the campaign. For example, there could be SBCC activities prior to the campaign, during and after the campaign. Some of the channels identified include the use of posters, community information centers, home visits, and in churches and mosques. This would ensure that communities are well sensitized before and after the campaign.

For peer review only - http://bmjopen.bmj.com/site/about/guidelines.xhtml

DISCUSSION

This study used a participatory process[18,19] to develop a framework (campaign transitioning strategy) that could transition LLIN distribution into the Community-based Health Planning and Services structure, which is the Primary Health Care system in Ghana. We actively involved various stakeholders to strengthen the development of an intervention that has the potential to systematically address real-world problems,[16] and to achieve sustainable outputs and impact [20,21] in malaria prevention and control. This would also help to achieve the global agenda of eliminating malaria.

From a practical point of view, this framework will shape the policy on LLINs distribution to ensure a continuous all year-round campaign on the regular use of LLINs. This would not only help address the challenges associated with periodic campaign rolled out during the PMD but ensure a reduction in the access to use gap. Another practical contribution of this framework would be the development of a guideline and training manuals on capacity building that support the transfer of CHAT to other communities. The use of local resources makes the intervention sustainable and easily integrated into the health care delivery system in Ghana. If adopted, the CHAT could become one of the flagship malaria prevention and control interventions which can contribute toward malaria elimination. The framework would also be suitable during public health emergencies that tend to disrupt facility-based service delivery.[22] Disruptions to malaria control programmes during public health emergencies have been linked to over 75 major resurgences arising from the 1930s through the 2000s.[23]

In summary, the present foundational study provides a framework for transitioning LLINs campaign into the primary health care system in LMIC settings. In doing so, it will add value to implementation science and practice concerning the scaling up and advancement of such

For peer review only

strategies to address challenges in LLINs campaigns. The next step is for investigators to assess the acceptability and feasibility of the CHAT campaign transitioning intervention. We are mindful of an obvious limitation which will be to consider contextual issues (malaria prevalence, social and cultural) that may relate to particular communities.

AUTHORS' CONTRIBUTIONS

All authors participated in designing the study with NYP and GMC providing technical support. PBA revised the final protocol. FG, EA and PTT collected and analysed the quantitative data while PDG was in charge of qualitative data collection and analysis. AN carried out the desk review. RH revised and finalized the CHAT framework. All authors participated in the Participatory Workshops. All authors contributed to writing the manuscript and approved of the final draft.

Competing Interests

The authors declare that they have no competing interests.

Funding

This work received financial support from the Health Campaign Effectiveness Programme, which is funded by the Bill & Melinda Gates Foundation at The Task Force for Global Health.

Data Sharing

Data are available upon reasonable request

For peer review only - http://bmjopen.bmj.com/site/about/guidelines.xhtml

REFERENCES

1. Allain JP, Owusu-Ofori AK, Assennato SM, et al. Effect of Plasmodium inactivation in whole blood on the incidence of blood transfusion-transmitted malaria in endemic regions: the african investigation of the mirasol system (AIMS) randomised controlled trial. Lancet. 2016;387(10029):1753–1761.

2. Ernst KC, Erly S, Adusei C, et al. Reported bed net ownership and use in social contacts is associated with uptake of bed nets for malaria prevention in pregnant women in Ghana. Malar J. 2017;16(1):13.

3. World Health Organisation (2019). World Malaria Report 2020

4. GSS/GHS/ICF Macro. *National Malaria Indicator Survey*. (2019).

5. Worrall, E., Were, V., Matope, A., Gama, E., Olewe, J., Mwambi, D., ... & Niessen, L. W. (2020). Coverage outcomes (effects), costs, cost-effectiveness, and equity of two combinations of long-lasting insecticidal net (LLIN) distribution channels in Kenya: a two-arm study under operational conditions. BMC public health, 20(1), 1-16. https://doi.org/10.1186/s12889-020-09846-4

6. Scott, J., Kanyangarara, M., Nhama, A., Macete, E., Moss, W. J., & Saute, F. (2021). Factors associated with use of insecticide-treated net for malaria prevention in Manica District, Mozambique: a community-based cross-sectional survey. *Malaria journal*, *20*(1), 1-9. https://doi.org/10.1186/s12936-021-03738-7

7. Aberese-Ako, M., Magnussen, P., Ampofo, G. D., & Tagbor, H. (2019). Health system, socio-cultural, economic, environmental and individual factors influencing bed net use in the prevention of malaria in pregnancy in two Ghanaian regions. Malaria journal, 18(1), 1-13. https://doi.org/10.1186/s12936-019-2994-5

8. Baltzell, K., Harvard, K., Hanley, M., Gosling, R., & Chen, I. (2019). What is community engagement and how can it drive malaria elimination? Case studies and

stakeholder interviews. Malaria journal, 18(1), 1-11. https://doi.org/10.1186/s12936-019-2878-8

9. Opoku, R., Amoah, P. A., & Nyamekye, K. A. (2021). Householders' perception about sustaining the useful life of long-lasting insecticide-treated nets in Ghana. International Health, 13(1), 57-62.

10. Bannor, R., Asare, A. K., Sackey, S. O., Osei-Yeboah, R., Nortey, P. A., Bawole, J. N., & Ansah, V. (2020). Sleeping space matters: LLINs usage in Ghana. *Pathogens and Global Health*, *114*(5), 271-278. https://doi.org/10.1080/20477724.2020.1776920

11. Assan, A., Takian, A., Aikins, M., & Akbarisari, A. (2018). Universal health coverage necessitates a system approach: an analysis of Community-based Health Planning and Services (CHPS) initiative in Ghana. Globalization and health, 14(1), 1-10. https://doi.org/10.1186/s12992-018-0426-x

12. Malede, A., Aemero, M., Gari, S. R., Kloos, H., & Alemu, K. (2019). Barriers of persistent long-lasting insecticidal nets utilization in villages around Lake Tana, Northwest Ethiopia: a qualitative study. BMC public health, 19(1), 1-11. https://doi.org/10.1186/s12889-019-7692-2

13. Wakefield, M. A., Loken, B. & Hornik, R. C. Use of mass media campaigns to change health behaviour. *The Lancet* vol. 376 1261–1271 (2010).

14. Dako-gyeke, P., Amazigo, U. V, Halpaap, B. & Manderson, L. Social innovation for health : engaging communities to address infectious diseases. *BMC Infect. Dis. Poverty* **9**, 1–4 (2020).

15. Nyonator, F. K., Awoonor-Williams, J. K., Phillips, J. F., Jones, T. C. & Miller, R. A. The Ghana Community-based Health Planning and Services Initiative for scaling up

For peer review only

service delivery innovation. *Health Policy and Planning* vol. 20 25–34 (2005).

16. Binka, F. N., Nazzar, A. & Phillips, J. F. The Navrongo Community Health and Family Planning Project. *Stud. Fam. Plann.* **26**, 121–139 (1995).

17. Awoonor-Williams, J. K. *et al.* The Ghana essential health interventions program: a plausibility trial of the impact of health systems strengthening on maternal & child survival. *BMC Health Serv. Res.* **13**, S3 (2013).

18. Loewenson, R. *et al.* Raising the profile of participatory action research at the 2010 Global Symposium on Health Systems Research. *MEDICC Rev.* **13**, 35–8 (2011).

19. McConnell, T. *et al.* Coproduction for feasibility and pilot randomised controlled trials: learning outcomes for community partners, service users and the research team. *Res Involv Engag.* **4**, (2018).

20. Jagosh, J. *et al.* Uncovering the benefits of participatory research: implications of a realist review for health research and practice. *Milbank Q.* **90**, (2012).

21. Leask, C., Sandlund, M., Skelton, D. & Chastin, S. Co-creating a tailored public health intervention to reduce older adults' sedentary behaviour. *Heal. Educ J* **76**, 595–608. (2017).

22. Agyepong, I. A. A Systems View and Lessons from the ongoing Ebola Virus outbreak in West Africa. *Ghana Med. J.* **48**, 168–172 (2014).

23. Walker, P. G. T. *et al.* Malaria morbidity and mortality in Ebola-affected countries caused by decreased health-care capacity, and the potential effect of mitigation strategies: a modelling analysis. *Lancet Infect Dis.* **15**, 825–832 (2016).

Figure 1: Images of Participatory Workshops held in the communities

Figure 2: Community Health Advocacy Team (CHAT) for LLIN Campaign Intervention

For peer review only - http://bmjopen.bmj.com/site/about/guidelines.xhtml

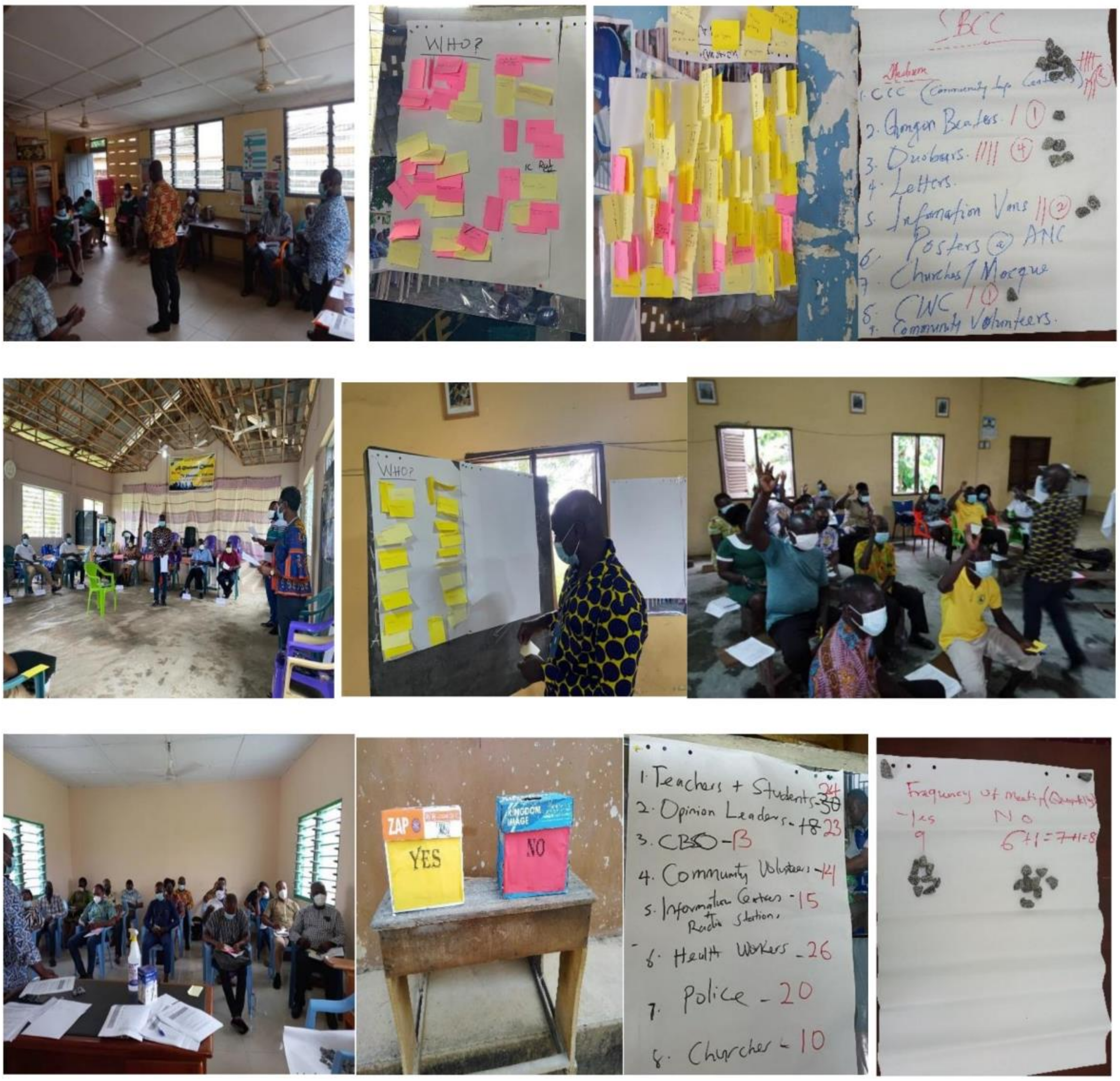

Figure 1: Images of Participatory Workshops held in the communities

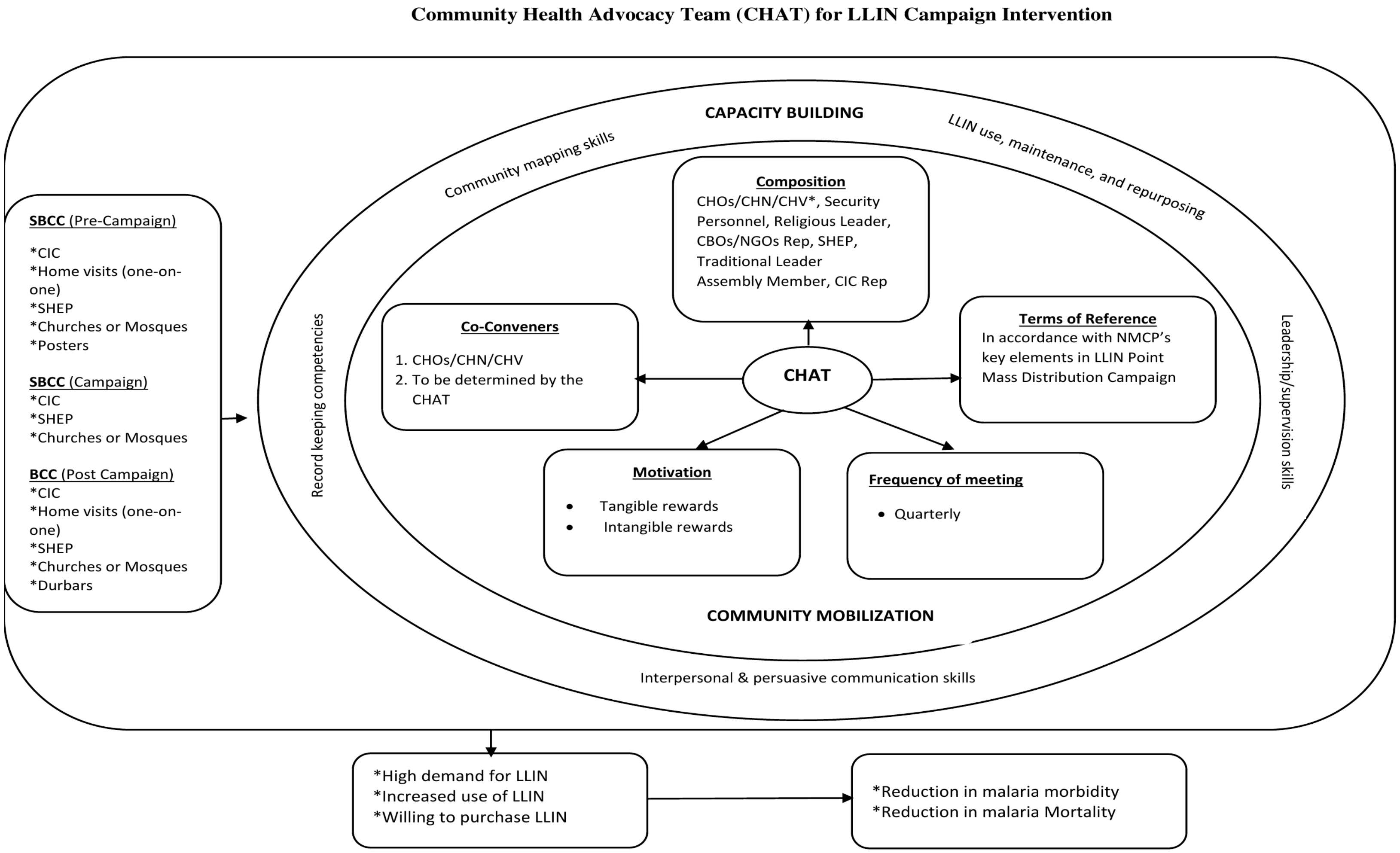

CHAT – Community Health Advocacy Team; SBCC – Social and Behaviour Change Communication; CIC – Community Information Centre; SHEP – School Health Education Programme; CHV – Community Health Volunteer, CHO – Community Health Officer; CHN – Community Health Nurse; CBO – Community Based Organization; NGO – Non-Governmental Organization

Figure 2: Community Health Advocacy Team (CHAT) for LLIN Campaign Intervention

APPENDIX

ITEM NO.	ITEM	DESCRIPTION	REPORTED ON PAGE NO.
1	Brief Name	The title or abstract identified of this social innovation in health research study.	1
2	Problem	Describe the current context, background and problem addressed by the social innovation from the perspective of the end user.	2
3	Rationale	Describe the rationale for the social innovation, including factors that show a change is needed from the perspective of the end user.	2-3
4	Social Innovation	Describe the key components of the social innovation. This could be accompanied by a detailed description, a photograph, or a figure. Describe each of the processes, activities, and elements used in the social innovation, including any enabling or supporting activities.	7-9
5	End Users	Describe the end users of the social innovation in health. Describe how end users are also direct or indirect beneficiaries of the social innovation.	8-9
6	Stakeholder Involvement	Describe how local stakeholders, including end users, are involved in design, development, implementation, and evaluation of the social innovation in health. In addition, describe the role of marginalized/vulnerable individuals or groups (e.g., people with disability or others as defined by the innovators) in these processes.	3-6
7	Inputs	Describe any physical, digital or informational materials used or distributed during training, delivery and/or implementation of in the social innovation; provide information on where the materials can be accessed† (e.g. online, appendix, URL).	5-6
8	Provider	For each category of the social innovation provider (e.g. community member, trained layperson, other individual), describe their expertise, background, role and any specific training given.	8-9
9	Implementation Strategy	Describe the implementation strategy for the social innovation and whether it is delivered individually, as a group, or partnership. Describe the level of external resources for implementation (e.g., internet access). Describe the frequency and duration of the social innovation delivery.	8-9

ITEM NO.	ITEM	DESCRIPTION	REPORTED ON PAGE NO.
10	Monitoring & Evaluation Strategy	Describe what is measured, how, and when as part of monitoring and evaluation. This includes measurement of health, social, and other impacts.	N/A
11	Setting	Describe the population, type(s) of location(s) where the social innovation is delivered, including any necessary social, political, cultural, environmental or other contextual issues. Describe at what level the innovation is implemented (e.g., district, subdistrict, village). This includes a description of the online setting for online social innovation.	N/A
12	Adaptability	Consider how the social innovation could be adapted, scaled up, or used in contexts other than the one described, if appropriate.	12
13	Financing	Describe how the social innovation in health has been funded at design, development, implementation, and evaluation stages. Describe how the social innovation could generate revenue (if applicable) or be institutionalized (if applicable) in order to be sustained in the future.	N/A
14	Health Impact	Describe the health impact of the social innovation over a period of time and the methods to assess health impact. Health is defined broadly here according to the WHO definition.	N/A
15	Social Impact	Describe the non-medical impact of the social innovation over a period of time. This could be impact on the environment, social changes, or other non-medical impact (e.g. lessons learned, new processes that emerged from the project, new relationships and networks, application of learned processes to other problems).	N/A
16	Limitations	Describe the limitations and potential unintended consequences of the social innovation in health during the design, development, or implementation.	12
17	Strengths	Describe how the social innovation in health improves on conventional practice.	12

SOCIAL INNOVATION IN HEALTH INITIATIVE

SESH Crowdsourcing For Health

TDR For research on diseases of poverty
UNICEF • UNDP • World Bank • WHO
